# Dr Sambhu Nath De: unsung hero

**Published:** 2011-02

**Authors:** G. Balakrish Nair, Yoshifumi Takeda

**Affiliations:** National Institute of Cholera & Enteric Diseases, Beliaghata, Kolkata 700 010, India; * Collaborative Research Center of Okayama University for Infectious Diseases in India, Beliaghata, Kolkata 700 010, India gbnair_2000@yahoo.com

His work sang but he was unsung. That is perhaps the best way to describe the discovery of cholera toxin by Dr Sambhu Nath De in Calcutta (now known as Kolkata). In a pioneering series of research spanning less than a decade starting from 1952 and culminating in the Nature paper of 1959^1^, De worked relentlessly in search of the elusive toxin that caused the epidemic responsible for the killer disease cholera. De’s discovery of cholera toxin was almost three quarters of a century after Robert Koch first cultured *Vibrio cholerae* (known as comma bacillus at that time) in Calcutta and 105 years after the microscopic observation of the causative agent of cholera by an Italian anatomist Filippo Pacini and the classic epidemiologic studies of John Snow, a British physician describing the waterborne nature of the disease. Prior to this, it was widely believed that the disease was caused by ‘miasma’ or unpleasant or unhealthy vapours emanating from sewers.

De’s method to understand the pathogenesis of cholera was distinctly different from all previous approaches. At that time, researchers believed that the pathogenesis of cholera was related to endotoxin and therefore used a multiplicity of methods and routes to replicate the disease which included introducing cholera stools into a variety of animals with little success. Following the course of the disease, De contended that the causative agent affects the permeability of the intestinal epithelium leading him to perform work at that interface using the now famous rabbit ileal loop model^2^. Not only was De able to replicate the disease with the causative organism introduced in the ileal loops of the rabbit but also was able to replicate the disease with bacteria-free culture filtrate of *V. cholerae* clearly showing that the exotoxin nature of cholera toxin. He also went on to perform experiments which led to the birth of the concept of enterotoxin producing *Escherichia coli* which today is firmly entrenched as enterotoxigenic *E. coli*^3^.

De’s discovery of cholera toxin introduced a new paradigm in research on cholera. A recent search done on November 19, 2009 in the PubMed database using the keyword “cholera toxin” yielded a phenomenal 11, 168 publications that the work of De spawned^4^. De’s work on cholera toxin has impinged into diverse areas such as cellular physiology, biochemistry and immunology. His work was clearly far ahead of his times. He did receive International recognition for this work including the nomination for the Nobel Prize by none other than Nobelist Professor Joshua Lederberg (Eugene Garfield, personal communication). But back home in India, his work remained unsung and unnoticed. The reasons for this oversight are described elsewhere^5^.

We usually end our lectures on cholera in India with two questions, who discovered the causative agent of the disease cholera and the other question is who discovered cholera toxin. Invariably there is an answer for question 1 but rarely do participants know the answer to question 2. We hope our efforts to extol the work of Dr Sambhu Nath De including this special edition of the Indian Journal of Medical Research would lead to a spontaneous answer on the person who devoted his research to understand the pathogenesis of cholera.
